# Surgical resection for hepatic metastasis from gastric cancer: a multi- institution study

**DOI:** 10.18632/oncotarget.16705

**Published:** 2017-03-30

**Authors:** Ailin Song, Xiaofeng Zhang, Feng Yu, Debang Li, Wenyu Shao, Yanming Zhou

**Affiliations:** ^1^ Department of General Surgery, Second Hospital of Lanzhou University, Lanzhou, China; ^2^ Department of Special Treatment, Eastern Hepatobiliary Surgery Hospital, Second Military Medical University, Shanghai, China; ^3^ Department of Hepatobiliary Surgery, the 101th Hospital of Chinese PLA, Wuxi, China; ^4^ Department of General Surgery, First Hospital of Lanzhou University, Lanzhou, China; ^5^ Department of Liver Surgery, First Affiliated Hospital of Nanjing Medical University, Nanjing, China; ^6^ Department of Hepatobiliary and Pancreatovascular Surgery, First Affiliated Hospital of Xiamen University, Xiamen, China

**Keywords:** gastric cancer, liver metastases, hepatectomy, prognosis

## Abstract

**Background:**

The beneficial effect of surgical resection for hepatic metastasis from gastric cancer (HMGC) remains elusive. This study was conducted to analyze surgical outcomes of HMGC and determine the prognostic factors associated with survival.

**Results:**

The in-hospital mortality rate was zero, and the overall morbidity rate was 56%. The overall 1-, 3-, and 5-year survival rate after surgery was 87.5%, 47.6%, and 21.7%, respectively, with a median survival time of 34.0 months. Multiple liver metastases (hazard ratio [HR] =1.998; 95% confidence interval [CI] = 1.248-3.198; *P* = 0.004) and ≥ T3 stage of the primary gastric cancer (HR = 2.065; 95% CI = 1.201–3.549; *P* = 0.009) were independent prognostic determinants in the multivariate analysis.

**Materials and Methods:**

Data on surgical resection of 96 patients with HMGC at six institutions in China were analysed retrospectively. Prognostic factors were assessed by multiple stepwise regression analysis using the Cox model.

**Conclusions:**

Surgical resection for HMGC is feasible and beneficial to long-term survival in selected patients.

## INTRODUCTION

It was reported that about 14% gastric cancer patients developed hepatic metastasis, and more than 30% gastric cancer patients developed metachronous liver metastasis after surgical resection of the primary gastric cancer [1]. Although previous studies [2–32] reported that hepatectomy may provide an opportunity of long-term survival in these patients (Table 1), most of these studies were conducted on the single-institution basis including no more than 50 cases, and therefore the beneficial effect of hepatectomy are not well-defined. The objective of this study was to analyze the outcome of surgical resection of hepatic metastasis from gastric cancer (HMGC) and determine prognostic factors associated with survival in a relatively large multi-institution cohort of patients.

**Table 1:** Literature overview of outcomes following hepatectomy for metastatic gastric cancer

Reference	Year	Country	Study interval	No. of patients	Mortality (%)	3-year OS (%)	5-year OS (%)	MST (months
Ambiru et al. [2]	2001	Japan	1975–1999	48	0	–	18	12
Imamura et al. [3]	2001	Japan	1990–1997	17	0	22	0	–
Okano et al. [4]	2002	Japan	1986–1999	19	0	34	34	–
Zacherl et al. [5]	2002	Austria	1980–1999	15	6.7	14.3	0	8.8
Sakamoto et al. [6]	2003	Japan	1985–2001	22	5	38	38	21
Shirabe et al. [7]	2003	Japan	1979–2001	36	0	26	26	–
Adam et al. [8]	2006	France	1983–2004	64	–	–	27	15
Sakamoto et al. [9]	2007	Japan	1990–2005	37	0	–	11	31
Cheon et al. [10]	2008	Korea	1995–2005	41	3	31.7	20.8	17
Morise et al. [11]	2008	Japan	1989–2004	18	0	27	27	13
Thelen et al. [12]	2008	Germany	1988–2002	24	4.2	22	15	10
Ueda et al. [13]	2009	Japan	1991–2005	15	0	60	60	–
Makino et al. [14]	2010	Japan	1992–2007	16	0	46.4	37.1	38.3
Tsujimoto et al. [15]	2010	Japan	1980–2007	17	0	37.5	31.5	34
Dittmar et al. [16]	2012	Germany	1995–2009	15	0	54	27	48
Garancini et al. [17]	2012	Italy	1998–2007	21	0	31	19	11
Miki et al. [18]	2012	Japan	1995–2009	25	–	42.8	36.7	33.4
Schildberg et al.[19]	2012	Germany	1972–2008	31	6	25	13	14
Takemur et al. [20]	2012	Japan	1993–2011	64	0	50	37	34
Wang et al. [21]	2012	China	2003–2008	30	0	16.7	16.7	11
Baek et al. [22]	2013	Korea	2003-2010	12	0	39	39	31
Chen et al. [23]	2013	China	2007–2012	20	0	20	–	22.3
Qiu et al. [24]	2013	China	1998–2009	25	0	70.4	29.4	38
Komeda et al. [25]	2014	Japan	2000–2012	24	0	40.1	40.1	22.3
Wang et al. [26]	2014	China	1996–2008	35	0	17.9	10.3	14
Kinoshita et al. [27]	2015	Japan	1990–2010	256	1.6	77.3	41.9	31.1
Shinohara et al. [28]	2015	Japan	1995–2010	19	0	31.7	31.7	27
Tiberio et al. [29]	2015	Italy	1997–2011	53	–	14	9.3	13
Oki et al. [30]	2016	Japan	2000–2010	94	–	51.4	42.3	40.8
Tiberio et al. [31]	2016	Italy	1990–2013	105	0.9	20.3	13.1	14.6
Markar et al. [32]	2017	UK	1997–2012	78	10.3	42	31	–

Abbreviations: MST = median survival time; OS = overall survival.

## RESULTS

### Patient characteristics

The study group included 72 (75%) men and 24 (25%) woman with a median age of 63 (range 32–78) years. Of these, 59 (61.5%) patients presented with synchronous liver metastases and 37 (38.5%) presented with metachronous liver metastases. In the latter group, the median interval between gastrectomy and hepatectomy for hepatic metastasis was 8.7 months (range, 4–32 months). Forty-two (43.7%) patients presented with solitary lesions, and 54 (56.3%) had multiple lesions. Ninety-one (94.8%) patients achieved curative resection. Fifty-eight (60.4%) patients received adjuvant therapy after hepetectomy.

### Results of surgical resection

There was no intra- or postoperative mortality. A total of 63 complications occurred in 46 patients (47.9%), of whom 11 (11.4%) patients had major morbidities (Clavien-Dindo ≥ 3a).

During the median follow-up period of 33 months, 77 (80.2%) patients experienced recurrences. The most frequently site of recurrence was the liver (*n* = 45, 58.4%), followed by the lymph node (*n* = 11, 14.3%), peritoneum (*n* = 8, 10.3%), and lung (*n* = 8, 10.3%). Details of the recurrences were unknown in 5 cases (6.5%). Only 3 (6.7%) of the 45 patients with liver recurrences underwent re-hepatectomy.

**Figure 1:**
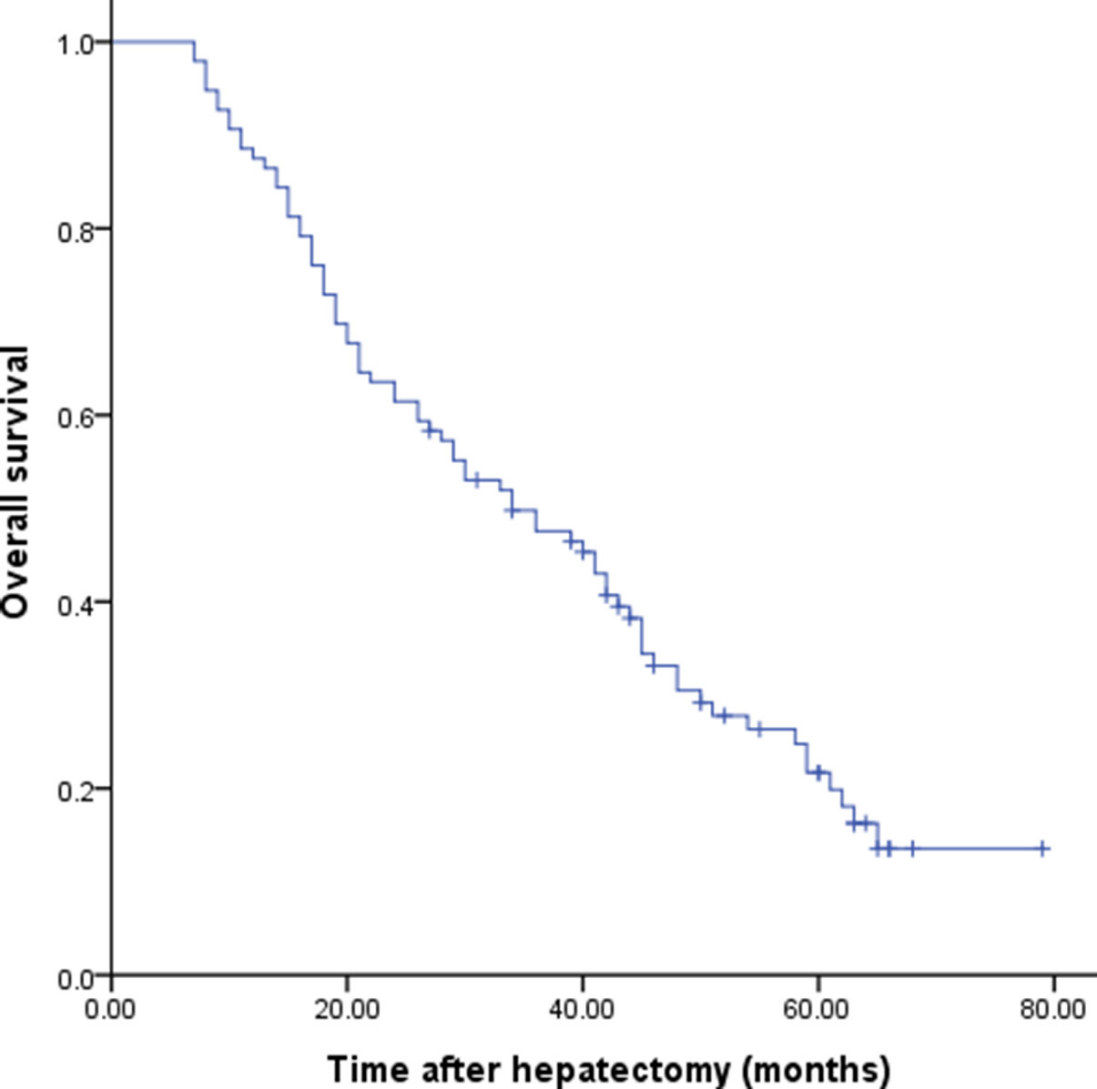
Cumulative overall survival for patients who underwent resection for hepatic metastasis from gastric cancer

The 1-, 3- and 5-year overall survival (OS) rate of the entire cohort of patients after surgery was 87.5%, 47.6% and 21.7%, respectively, with a median survival time of 34.0 months (Figure 1). Several factors were found to be associated with poor prognosis in univariate analysis (Table 2). Multiple liver metastases (hazard ratio [HR] = 1.998; 95% confidence interval [CI] = 1.248–3.198; *P* = 0.004) and ≥ T3 stage of the primary gastric cancer (HR = 2.065; 95% CI = 1.201–3.549; *P* = 0.009) were independent prognostic determinants, as shown by multivariate analysis.

**Table 2:** Univariate analysis of prognostic factors for overall survival

Variables	Number	MST (months)	*P*-value
Gender			
Male	72	40	0.714
Female	24	28	
Age			
≥ 65	51	36	0.739
< 65	45	34	
T stage of gastric cancer			
< T3	47	46	0.007
≥ T3	59	24	
Lymph node status of gastric cancer			
N0-N1	28	45	0.015
N2-N3	68	30	
Histologic grade of gastric cancer			
Well-moderate	62	42	0.032
Poorly	34	29	
Histologic type of gastric cancer			
Intestinal	61	44	0.058
Diffuse	12	17	
Type of gastrectomy			
Total gastrectomy	33	22	0.373
Subtotal gastrectomy	63	40	
Timing of hepatic metastasis			
Synchronous	59	43	0.167
Metachronous	37	33	
Type of hepatectomy			
Major (≥ 3 segments)	35	34	0.456
Minor (< 3 segments)	61	36	
Curative resection			
Yes	91	39	0.031
No	5	27	
Maximum hepatic metastasis size			
< 5 cm	71	44	0.069
≥ 5 cm	25	28	
Number of metastases			
Solitary	42	42	0.021
Multiple	54	29	
Distribution of liver metastases			
Unilobar	57	41	0.394
Bilobar	29	32	
Morbidity			
Yes	46	40	0.237
No	50	31	
Adjuvant chemotherapy			
Yes	58	42	0.172
No	38	35	

Abbreviations: MST = median survival time.

## DISCUSSION

Although solid clinical evidence exists to support surgical resection as the optimal option for the treatment of colorectal liver metastasis (CLM), data evaluating the postoperative outcome of HMGC are limited and controversial (Table 1). This may be explained by the fact that most patients with HMGC presented with multiple lobar metastases, gross peritoneal dissemination, extensive lymph node metastases, distant metastasis, or direct invasion to other organs at time of diagnosis, and therefore were considered unamenable to surgery [5]. It was reported that patients with potentially resectable HMGC treated with systemic chemotherapy had a median survival of 5.5 months, and the number of 3-year survivors was zero [23]. A systematic review of 870 patients from 23 studies performed by Petrelli and colleagues in 2015 demonstrated that resection of HMGC was associated with a 22-month median survival and a 23.8% 5-year OS rate [33]. A similar systematic review was published by Markar et al in 2016, which included 991 patients and come to similar conclusions, with a median 5-year OS of 27% [34]. In accordance with these two reports, the present study also confirmed the benefit of surgical resection for HMGC in achieving long-term survival in a relatively large cohort of patients drawn from multi-centers in China. The 5-year OS rate after surgery was 21.7% with a median survival time of 34.0 months.

Safety is an important concern in such an aggressive management strategy. Hepatic resection of metachronous liver disease is technically demanding because of intra-abdominal adhesions caused by previous surgery. Synchronous resection of the HMGC and primary tumor may be associated with increased post-operative complications. However, in the present study and previous reports, resection could be safely performed with acceptable operative mortality and morbidity [19–28]. It should be noted that this favourable outcome may be attributed to careful selection of patients with limited disease, as reflected by major hepatectomy is not performed frequently [27]. In addition, patients with HMGC usually had no underlying cirrhosis, a factor associated with a high risk of postoperative liver failure because of the limited regenerative capacity of the liver remnant.

Multivariate analysis in our series disclosed that multiple liver metastases and ≥ T3 stage of the primary gastric cancer were independent prognostic determinants. These data may have important implications in helping select patients for surgical resection. There is evidence that postoperative complications, especially major complications, have a negative impact on long-term survival after oncological surgery [35]. Immunosuppression secondary to septic complications may increase the growth of occult micrometastasis, and some complications may make patients unfit for adjuvant therapy or delay the time interval. However, we did not find that OS was significantly reduced in patients with complications in our study. The fact that there were only 11.4% of patients with major complications in current study, it is possible that failure to detect a clinically significant difference represents a type-II error.

Percutaneous radiofrequency ablation (RFA) is generally accepted as a safe, effective and minimally invasive treatment for patients with CLM [36]. However, few studies have focused on RFA for HMGC. RFA may contribute to local control of single liver-only metastatic lesions [37]. No reliable data on the comparative therapeutic efficacy of RFA and hepatic resection for resectable HMGC are available at present, and therefore RFA should not be recommended as an alternative to surgical resection at this disease stage.

Recurrence following surgical resection of HMGC is common and mostly occurs in the remaining liver tissue. Repeat hepatectomy seems beneficial to patients with solitary intrahepatic recurrence [25, 27]. Unfortunately, very few patients are potential candidates for hepatic resection because of extensive metastases [27].

The efficacy of adjuvant chemotherapy after liver resection of HMGC remains unclear. Qiu et al. [24] reported that patients who received adjuvant chemotherapy had significantly better survival. However, our and other studies [9, 25, 27] were unable to confirm their conclusion. These different findings may be explained by differences in the regimens used, the timing and duration of cytotoxic agent administration, and patient selection between these studies. Unlike CLM, it seems impossible to test the clinical significance of adjuvant chemotherapy for HMGC in a prospective manner because of the rarity of candidates [27].

The study has some limitations, including the retrospective nature and possible bias in patient selection. More data are needed to further verify the conclusion of the present study.

In conclusion, this large-cohort study has demonstrated that surgical resection for HMGC is feasible and beneficial to long-term survival in selected patients.

## MATERIALS AND METHODS

### Patients

Included in this study were 96 patients who underwent surgical resection for HMGC between January 2001 and January 2012 in six medical institutions in China. The study was approved by the ethics committee of each contributing institution. Written informed consent forms were not required from the patients due to the retrospective approach of the study. In general, surgical resection of HMGC followed the following criteria: (1) curative resection (microscopic tumor removal based on the histopathologic evaluation) of the primary tumor and liver metastasis thought to be technically accessible; and (2) no sign of concomitant extrahepatic metastasis on preoperative imaging. Data were collected retrospectively by reviewing the medical records, including patient age and sex at hepatic resection; pathological characteristics of the primary gastric cancer and hepatic metastasis; and short- and long-term outcomes after surgery. Postoperative mortality was defined as any death occurring within 30 days of surgery or within the same hospital stay. Postoperative complications were defined as occurrence of any medical or surgical complication during the hospital stay and graded according to the Clavien-Dindo classification [38]. The primary tumor stage and regional lymph node status were classified according to the 7th edition of the International Union Against Cancer of gastric cancer [39]. The types of hepatectomy were classified in accordance with the Brisbane 2000 nomenclature [40].

### Statistical analysis

Overall survival was determined by Kaplan-Meier analysis. Factors related to survival selected by univariate analysis with *P* < 0.05 were entered into a multivariate analysis using Cox proportional hazard regression model to determine the independent risk factors for survival. All statistical analyses were performed using SPSS for Windows (version 11.0; SPSS Institute, Chicago, IL, USA). *P* < 0.05 was considered statistically significant.

## Abbreviations

HMGC: hepatic metastasis from gastric cancer
OS: overall survival
HR: hazard ratio
CI: confidence interval
CLM: colorectal liver metastasis
RFA: radiofrequency ablation
